# Supplementary material for: Association between care burden, depression and personality traits in Alzheimer’s caregiver: A pilot study
Source: PLoS One. 2021 Sep 29;16(9):e0251813. doi: 10.1371/journal.pone.0251813 (PMC8480609; doi:10.1371/journal.pone.0251813)
Supplement: S1 Appendix — (DOC) [file pone.0251813.s001.doc]

S1 Appendix

**SASB - Structural Analysis of Social Behaviours - Rules for determining Intrapsychic Behaviours**: (inward attention): attention is turned inwards. Transitive action with the person himself as the subject and at the same time as object. This form can be either active or passive. The person does things for himself or thinks of something concerning oneself.

Interpersonal: (attention towards the other): attention is turned outwards, doing something for, or with reference, to others. This does not include what others do for the person, but implies an action involving the other as the direct object: an action is expressed influencing someone or something and a direct object is needed to complete the sequence. Actions start with a proposer and have an effect on someone or something.

Attention towards the other can be either active or passive: the phrase “ you hit me” and “I was hit by you” are equivalent SASB evaluations. In both cases the action is expressed by the one same person and results in affecting the other.

1- Inward attention – 8 clusters

**1.** Assertive and separating. This type does what seems right on the basis of what he considers is necessary at the time. The attitude may be spontaneous, with self-acceptance, and pleasure in the experience. Or tt could be disoriented and the person give little weight to problems and important choices in life.

**2.** Self-accepting and exploring. This type accepts and reacts to his deepest feelings, feeling solid, integrated and “together”. The desire to be open to feelings generally indicates a state of self-satisfaction and acceptance of weak and strong points.

**3.** Self-supporting and appreciative. This type is deeply appreciative of himself and is able to treat, care for, console and reconsolidate himself. He has a capacity for self-esteem and in extreme cases of self-adoration.

**4.** Self-care and development. This type protects and realistically examines himself and has the capacity to be positively self-constructive, actively developing his abilities and other important qualities for self-growth. This can imply using much energy to obtain what is needed and desired.

**5.** Self-regulating and controlling. This type can control himself. Great self-control is exercised for chosen objectives. This may include paying attention to behaviour in order to ensure conforming to ideals, including great activity programmed to reach objectives.

**6.** Self-critical and oppressive. This type oppresses himself and may accuse himself of inadequacy, evoking feelings of self-guilt and shame. Feelings of uncertainty and guilt can be used for false induction to what is recognized as not being useful to the person. This could be self-punitive behaviour, sometimes destructive enough as to call for therapeutic intervention.

**7.** Self-refusing and annulling. This self-destructive type may ignore illness and wounds, exhaust himself and become completely listless. This implies self-refusal and self-deprivation and generally self-inflicted cruelty. Such self-destructive behaviour calls for serious qualified psychotherapeutic intervention.

**8.** Self-negligent and mentally absent. This type may daydream, subsequently not developing his abilities and potentials to the full. In extreme cases he may have unreasonable and unjustified ideas about himself and behave without any criterion, falling into self-destructive situations. In these cases it could be beneficial to examine the danger of self-destructive behaviour with a therapist.

2- Attention toward the other – 8 clusters

**1.** Liberating and forgetting. The type emancipates, liberates the other by expressing trust and encouraging his independent identity. However this emancipation may include elements of neglect and forgetfulness.

**2.** Confirming and understanding. This type is appreciative, understanding and confirming, being empathic toward the other. Behaviour patterns which may also be present include treating the other justly, listening to him attentively even if there are differences of opinion.

**3.** Caring and consoling. This type is described as caring, attentive, consoling and desiring to be close to the other person.

**4.** Helping and protecting. This type is described as someone who actively helps the other by protecting, sustaining, advising and even teaching him. This kind of help could, if extreme, turn to spoiling and indulging behaviour.

**5.** Looking after and managing. This type controls the other, reminding him what should be thought, done and said, for the “good of the other person”. Other less positive forms of control can be expressed as limiting freedom and forcing the other to conform to specific rules or orders.

**6.** Belittling and blaming. This type is described as belittling, blaming or manipulating the other in a deceitful way. He may try to make the other admit to faults and in extreme cases demand revenge and threaten with punishment.

**7.** Assaulting and refusing. This type can seriously threaten or hurt the other. Extreme behaviour may include physical or moral assault with destructive intention. Other forms might be: stinginess with necessities, ignoring, refusal with anger or taking advantage of the other person.

**8.** Ignoring and forgetting. This type is described as basically ignoring and neglecting the needs and interests of the other. This implies not attention and can produce or derive from unusual concepts and perceptions. Such treatment of the other can appear illogical and senseless. This type could abandon the other in more critical moments.
