# Supplementary material for: Association between care burden, depression and personality traits in Alzheimer’s caregiver: A pilot study
Source: PLoS One. 2021 Sep 29;16(9):e0251813. doi: 10.1371/journal.pone.0251813 (PMC8480609; doi:10.1371/journal.pone.0251813)
Supplement: Attachment — Submitted filename: Response to Reviewers.doc [file pone.0251813.s002.doc]

Dear Editor and Reviewers

I hope I have fully answered the requests of both the Editor and the Reviewers.

Thank you for your comments.

I am especially grateful for the suggestions very useful for future investigations concerning the relationship between personality, care burden and quality of life of caregivers of patients with Alzheimer's dementia.

Best regards

Anna Vespa

Response to Editor and Reviewers.

1. *Please ensure that your manuscript meets PLOS ONE's style requirements, including those for file naming. The manuscript has been corrected based on PLOSONE’s style requirement*

The manuscript has been corrected based on your indications.

2. *We suggest you thoroughly copyedit your manuscript for language usage, spelling, and grammar. If you do not know anyone who can help you do this, you may wish to consider employing a professional scientific editing service Acknowledgment to Monica Gleboki for her correction of the English.*

English was corrected (highlight in yellow)

3. *In line with PLOS' guidelines regarding the description and reproducibility of methods (please provide further detail on how the questionnaires and testing procedures were administered.*

The informations required have been included in the paragraph “materials and methods”.

…The caregivers were interviewed by two psychotherapists, working in the hospital, with one year training in learning and administering the SASB Model, in particular the Form-A. The caregivers filled out the questionnaires (which included socio-demographic and clinical variables) during the interview.

English was corrected.

4. *We note that you have indicated that data from this study are available upon request. PLOS only allows data to be available upon request if there are legal or ethical restrictions on sharing data publicly.*

The data can be requested directly from the Chief of the Department of Neurology Dr . Giuseppe Pelliccioni and from the Scientific Direction, IRCCS INRCA, motivating the same request. ([g.pelliccioni@inrca.it](mailto:g.pelliccioni@inrca.it); direzione scientifica@inrca.it)

5. *Please include captions for your Supporting Information files at the end of your manuscript, and update any in-text citations to match accordingly. Please see our*

Supporting information was included at the end of manuscript.

*- Please improve statistical reporting and ensure that numbers are properly formatted, i.e., with a decimal point instead of a comma.*

Numbers are properly formatted, i.e., with a decimal point instead of a comma (Text and Tables).

6. *Thank you for submitting the above manuscript to PLOS ONE. During our internal evaluation of the manuscript, we found significant text overlap between your submission and the following previously published works:*

*("Caregiver burden characterization in patients with Alzheimer's disease or vascular dementia", 2014, by D'Onofrio et al.)*

*We would like to make you aware that copying extracts from previous publications, especially outside the methods section, word-for-word is unacceptable. In addition, the reproduction of text from published reports has implications for the copyright that may apply to the publications.*

*Please revise the manuscript to rephrase the duplicated text, cite your sources, and provide details as to how the current manuscript advances on previous work. Please note that further consideration is dependent on the submission of a manuscript that addresses these concerns about the overlap in text with published work.*

*We will carefully review your manuscript upon resubmission, so please ensure that your revision is thorough.*

Some parts have been changed:

**The Caregiver Burden Inventory (CBI)** [25] is a 24 items tool for assessing the care burden, able to analyze the multidimensional, elaborate aspects of the caregiver’ s experience of patients with Alzheimer's disease and related dementias.

The CBI is a self-report tool, compiled by the principal caregiver, i.e. the family member or operator who most bears the burden of care. Divided into 5 sections, it allows to evaluate the following different stress factors: 1) Objective burden (items 1–5)- time dependent: the burden in relation to the time that the caregiver has to devote to his family member; 2) Developmental burden: the caregiver's perception of feeling cut off from expectations and opportunities of their peers (items 6–10); 3) Physical burden: the feelings of chronic fatigue and somatic health problem (items 11–14);s; 4) Social burden: the perception of a role conflict (items 15–19); 5) Emotional burden: the feelings towards the patient, that can be induced by unpredictable and bizarre behavior (items 20–24).

There are 5 items for each dimension, except for the physical burden, which has four items.

Therefore the total score for Time-dependence, Developmental, Social and Emotional burden range from 0 to 20 except for physical burden where scores range from 0 to 16. However, as suggested the score on physical burden also could be multiplied by 1.25 to obtain an equivalent score out of 20. The total score range ranges from 0 to 96: a score> 36 indicates a risk of "exhaustion" while scores close to or slightly above 24 indicate the need to seek some form of respite care.

**The Mini‐Mental State Examination(**MMSE) [28, 29] is the most commonly used dementia screening tool. The MMSE consists of 30 questions and includes tests of orientation, concentration, attention, verbal memory, naming and visuospatial skills. Scores for items are summed together into a total score. Higher scores indicate better cognition and the maximum score is 30. The total score, given by the sum of the scores that the patient has obtained for each item, can range from a minimum of 0 (maximum cognitive impairment) to a maximum of 30 (no cognitive impairment). The cutoff score is 23-24, and most non-demented older people rarely score below 24.

***Reviewers***

***Reviewer 1***

*Nevertheless, there were some interesting findings. One weakness of the researchers' introduction of the SASB model is that they do not explain the model or the three underlying dimensions and how the assessment of individuals (self or observers) work.*

Some parts in the introduction and discussion have been changed and expanded.

English was correct

The part required on the SASB has been introduced in the text and in the methodology:

For this reason, in this study we have implemented the SASB circumplex model used to describe personality from normal to pathology and defines interpersonal and intrapsychic behaviors by three underlying dimensions: focus (other, self, introject), affiliation-hostility (love-hate), and interdependence-independence (enmeshment-differentiation). SASB applications extend from research to practice: assessment, treatment of intrapsychic problems and verification of psychotherapeutic intervention[18, 19].

.

What were the reasons from the caregivers given for not participating?

It was explained in the methodology .

• What was the training of the psychologists on the SASB assessment surveys?

Were the psychologists part of the research team or employees of the hospital?

It has been specified in the methods paragraph:

The caregivers were interviewed by two psychotherapists, working in the hospital, with one year training in learning and administering the SASB, in particular the Form-A.

How were they recruited?

Seventy seven caregivers, meeting all inclusion criteria, were consecutively recruited at the Neurology Departments of INRCA-IRCCS National Institute of Science and Health in Ancona, Italy and asked to participate in the study. After completing this initial medical examination they were referred to the investigator

• An explanation of the SASB Model and its three underlying dimensions is important to include.

For this reason, in this study we have implemented the SASB circumplex model used to describe personality from normal to pathology and defines interpersonal and intrapsychic behaviors by three underlying dimensions: focus (other, self, introject), affiliation-hostility (love-hate), and interdependence-independence (enmeshment-differentiation). SASB applications extend from research to practice: assessment, treatment of intrapsychic problems and verification of psychotherapeutic intervention[18, 19].

• There are sections in the manuscript that needs significant editing. Some phrases are a bit confusing but understandable. The authors need to look at the document again…………

The text has been clarified, changed, corrected and expanded (changes highlighted in yellow).

The citations were introduced (page 8).

Reviewer 2

*The discussion/conclusions focus only on the correlations between the global outcomes for key measures which appear to be based on the whole sample rather than subsets as described in the tables. The authors have not clarified this discrepancy in how the data is presented.*
Some of these issues have been clarified and deepen in the discussion session.

I suggest that the paper be rewritten after sample size justifications have been determined and focus only on the data which can be statistically supported by the method. This may still yield valuable outcomes and conclusions about the relationship between personality and burden.

There was an error in Tables 1 and 2 now corrected.

The table shows the correct description of the sample. The sample size justifications have been determined and the data are statistically supported.
